# A novel variant c.A527G in *ITGB4* leads to autosomal dominant epidermolysis bullosa in China

**DOI:** 10.3389/fmed.2025.1726208

**Published:** 2026-02-05

**Authors:** Juyi Li, Xiawen Yang, Yimin He, Peng Cheng, Hengfei Li, Aiping Deng, Xiufang Wang, Wei Cai, Jifa Hu, Qiu Tang, Ying Gao, Yi Hu

**Affiliations:** 1 Department of Pharmacy, The Central Hospital of Wuhan, Tongji Medical College, Huazhong University of Science and Technology, Wuhan, Hubei, China; 2 Department of Dermatology, The Central Hospital of Wuhan, Tongji Medical College, Huazhong University of Science and Technology, Wuhan, Hubei, China; 3 Department of Infectious Diseases, Hubei Provincial Hospital of Traditional Chinese Medicine, Wuhan, Hubei, China; 4 Department of Pain, The Central Hospital of Wuhan, Tongji Medical College, Huazhong University of Science and Technology, Wuhan, Hubei, China; 5 Department of Gastrointestinal Surgery, The Central Hospital of Wuhan, Tongji Medical College, Huazhong University of Science and Technology, Wuhan, Hubei, China; 6 Department of Scientific Research, The Central Hospital of Wuhan, Tongji Medical College, Huazhong University of Science and Technology, Wuhan, Hubei, China; 7 Department of Oncology, The Central Hospital of Wuhan, Tongji Medical College, Huazhong University of Science and Technology, Wuhan, Hubei, China; 8 Department of Pulmonary and Critical Care Medicine, The Central Hospital of Wuhan, Tongji Medical College, Huazhong University of Science and Technology, Wuhan, Hubei, China

**Keywords:** autosomal dominant inheritance, epidermolysis bullosa, genetics, ITGB4, whole-exome sequencing

## Abstract

**Objective:**

This study aimed to uncover the genetic variations and their corresponding clinical features in a Chinese family affected by epidermolysis bullosa (EB).

**Methods:**

We enrolled a Chinese family clinically diagnosed with EB and conducted whole-exome sequencing on the proband to identify genetic variations. I-TASSER and PyMOL software were used to examine the structural and functional implications of the identified mutant proteins.

**Results:**

The study identified an autosomal-dominant form of epidermolysis bullosa simplex (EBS) in the family, attributed to a novel missense variation c.A527G (D176G) in the *ITGB4* gene. By bioinformatics analyses, we found that the wild-type D176 forms one hydrogen bond with a distance of 3.1 Å from F201, one hydrogen bond with a distance of 2.7 Å from K177, and two hydrogen bonds with a distance of 3.2 Å from Y304; however, the mutant G176 only forms one hydrogen bond with F201 at a distance of 3.2 Å.

**Conclusions:**

This study confirms the dominant mode of inheritance of the missense *ITGB4* mutation observed in EB. The novel missense variation c.A527G (D176G) in *ITGB4* involves a transition from a polar to non-polar amino acid and a decrease in intermolecular hydrogen bonding, which was associated with EB development.

## Introduction

Hereditary epidermolysis bullosa (EB) is a group of hereditary skin diseases caused by genetic variations in the adhesion complex of multiple structures in the basement membrane zone (BMZ) of the skin. It is characterized by easy blistering and erosion of the skin and mucosa after minor mechanical trauma (1, 2). These variations affect the adhesive structure of the skin, causing damage to the connections between skin layers, resulting in the formation of blisters (3).

The clinical manifestations of EB greatly vary, ranging from mild to severe fatal forms. In the mildest form of EB, patients may only present with pigmentary abnormalities and/or nail dystrophy without blisters or erythema. However, in the most severe cases, EB may be fatal during the perinatal or early postpartum period. These cases typically involve a wider range of skin and mucous membranes, which can lead to severe infections, malnutrition, dehydration, and electrolyte imbalances, as well as respiratory and digestive system complications caused by extensive skin loss (4, 5).

The diagnosis of EB is usually based on family history, clinical symptoms and signs, as well as histopathological examination via skin biopsy (6). In some cases, mutational analysis may also be necessary to determine specific genetic variations (7). The treatment is mainly supportive, including careful wound care, trauma avoidance, infection control, and pain management (8). For some severe cases, a multidisciplinary approach, including dermatologists, genetic counselors, nutritionists, and pain management specialists, may be necessary (9).

The ITGB4 protein is an important component of the BMZ of the skin, and together with other proteins such as the integrin alpha-6 subunit (ITGA6), it forms the integrin α6β4 complex. This complex is crucial for maintaining adhesion between the epidermal and dermal layers of the skin. It mediates cell adhesion to the basement membrane by interacting with laminin, thereby playing a critical role in the stability and integrity of the skin (10).

The types of EB are mainly defined based on the location the structural damage on the skin. There are currently four known types of EB: (1) epidermolysis bullosa simplex (EBS), herein, separation of vesicular tissue occurs in the epidermal basal keratinocytes adjacent to the basal layer; (2) junctional epidermolysis bullosa (JEB), blisters occur in the transparent layer of the basement membrane and are usually more severe; (3) dystrophic epidermolysis bullosa (DEB), blisters occur in the dense lower layer; and (4) Kindler syndrome (KS) (11, 12).

So far, EB caused by variations in the *ITGB4* gene is often found to be inherited in an autosomal recessive manner (13). However, there are also reports that the variations caused by *ITGB4* are dominant genetic diseases (14). Here, we report an autosomal dominant genetic variation caused by structural variations in *ITGB4* associated with JEB.

## Materials and methods

All candidates who participated in this project carefully filled out detailed questionnaires about their family history and history of their own diseases, and supplemented the collected information in their medical records. For the diagnosis and classification of EB, the whole research team strictly followed the latest international professional guidelines issued to conduct the work. All participants in this study signed the informed consent form after they were fully briefed on the purpose, process, potential risks, and expected benefits of the study. Furthermore, the study was approved by the Ethics Committee of Wuhan Central Hospital from the preparation to the execution of the study.

### DNA extraction and whole-exome sequencing (WES)

We acquired the clinical data from patients and their family members. We collected 5 ml of fasting blood samples using anticoagulant tubes containing EDTA from each person and isolated the genomic DNA using a DNA Extraction Kit (Beijing Tiangen Biotechnology Co., Ltd., China) according to the kit instructions. The obtained DNA was subjected to exon sequencing using a Sure Select Human All Exon V5 kit on the Illumina HiSeq2500 system to determine potential pathogenic genetic variants (15).

### Molecular genetic analyses

Single nucleotide polymorphisms (SNPs) and insertion deletion (InDels) variants were identified by comparing the obtained genome sequences with the reference sequence of the human genome and by comparing them with public databases including 1,000 Genomes, ExAC, dbSNP, ESP, and gnomAD. Variations that were not detected in the database were analyzed in depth to determine if they result in a change in the genetic code and to distinguish between synonymous and missense variations. Subsequently, computational biology tools (SIFT, Polyphen2, Mutation Taster, LRT, and FATHMM) were used to assess the possible effects of these non-synonymous variations on protein structure and function (16, 17).

### Sanger sequencing

Using Sanger's sequencing to identify genetic variations in patients and their families, specific primers were designed based on the mutation location in the *ITGB4* gene. The *ITGB4* primer sequence was as follows: forward primer, 5′- TATCCCCTCTCTGTCCTTT-3′ reverse primer, 5′- TGGGTGAAAGAGGAGTGGG-3′. The target DNA fragments were amplified by polymerase chain reaction (PCR) and sequenced using an ABI3730XL automated sequencer (Applied Biosystems, Foster City, CA). The sequencing results were compared with reference genome sequences obtained from a gene bank database. This comparative analysis was conducted using AutoAssembler 2.0.

### Structural modeling

The amino acid sequence of the ITGB4 protein was obtained from the NCBI database and the three-dimensional (3D) structure of the ITGB4 protein was simulated using the I-TASSER (https://seq2fun.dcmb.med.umich.edu//I-TASSER/) website. The PyMOL (https://pymol.org/2/) software was then used to analyze the structural differences between the wild-type and mutant proteins, and the changes in amino acid residues were observed and recorded.

## Results

### Clinical characteristics

**Figure 1:**
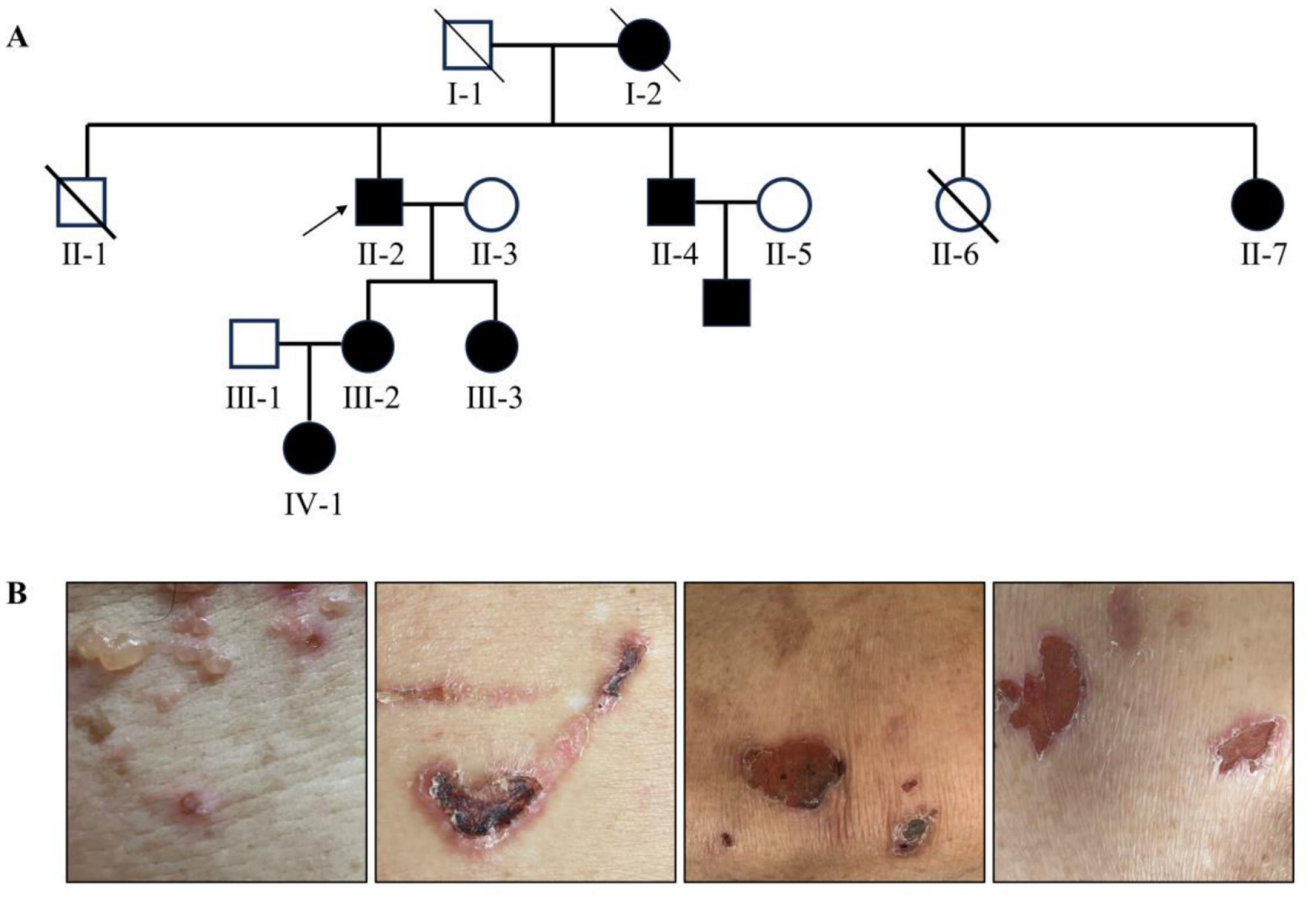
**(A)** Family pedigree. The square represents males and while the circle represents females. The affected family members are represented by black symbols, and the proband is represented by arrows. **(B)** Photographs of the upper and lower limbs of the proband.

The family pedigree of the proband II-2 is shown in Figure 1A. The proband's mother (I-2), younger brother (II-4), younger sister (II-7), two daughters (III-2, III-3), and granddaughter (IV-1) all exhibited clinical symptoms of EB. The affected members showed clinical symptoms of EB in both the upper and lower extremities, belly, and back (Figure 1B), and which developed blisters on the surface of the skin, with the skin lesions being the shallowest and not involving the mucosa or extracutaneous complications. Generally, there were no scars left after recovery. All these affected individuals had clinical symptoms of EB after birth. When they slept on a hard or cold bed, their skin would rupture; however, the above mentioned conditions would improve, and the broken skin could heal on its own.

### Genetic and bioinformatics analyses

**Figure 2:**
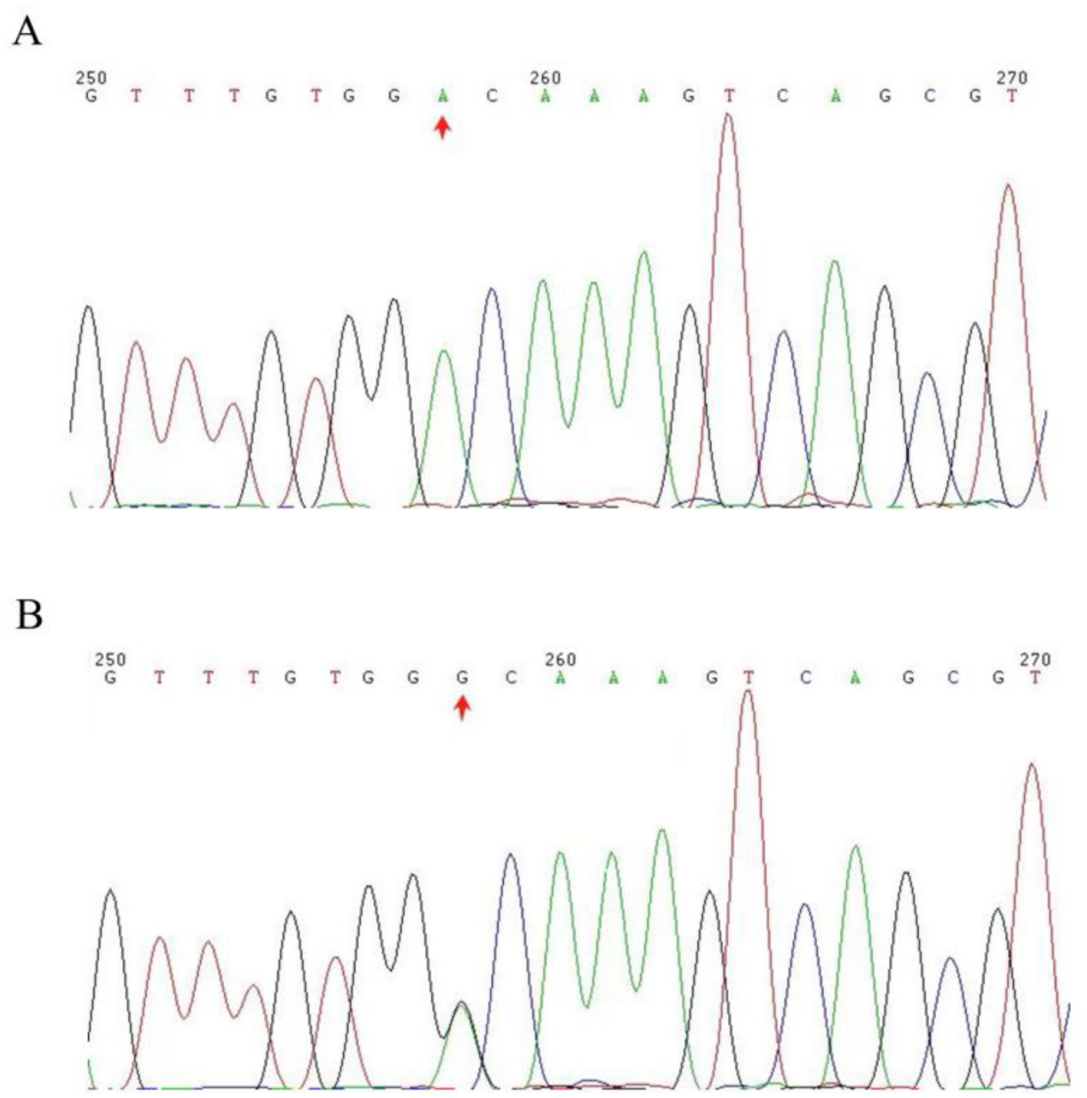
The chromatograms of the DNA sequencing. The arrows highlight the altered location of the mutation within the *ITGB4* gene. **(A)** Represents the *ITGB4* gene in its original form. **(B)** shows the *ITGB4* gene with a heterozygous mutation.

In all, 141,885 variants were found by WES of the proband (II-2), including 127,172 single nucleotide polymorphisms (SNPs) and 14,713 insertion-deletions (InDels; Table 1). A panel of pathogenic genes related to EB (*CD151, COL7A1, COL17A1, DSP, DST, EXPH5, FERMT1, ITGA3, ITGA6, ITGB4, KLHL24, KRT14, LAMA3, LAMB3, LAMC2*, and *PLEC*) were first analyzed. *ITGB4* was found to be the pathogenic gene in the proband, and the variant was located on chromosome 17, position 73724515, *ITGB4*:NM_001005619:exon5: c.A527G:p.D176G. The variant was not record in any databases (1,000 g, esp6500 and GnomAD); therefore, the variant was deemed novel. Multiple bioinformatics prediction software (SIFT:0.0; Polyphen2_HVAR:0.982; Polyphen2_HDIV:0.999; MutationTaster:1; LRT:0.000; and FATHMM:-4.88; REVEL:0.984) revealed that the variant was harmful and damaged the protein function. Through Sanger sequencing, it was found that the novel variant was present in the affected members (II-2, II-4, III-2, and III-3), but not in the other healthy relatives II-3 and III-1 (Figure 2). The novel variant (NM_001005619: c.A527G:p.D176G) in *ITGB4* was classified as likely pathogenic (PM1+PM2+PP1+PP2+PP3+PP4).

**Table 1:** Whole-exome sequencing detail of the patient.

**Exome capture statistics**	**Patient**
Total (bp)	94,254,524 (100%)
Duplicate (bp)	15,014,770 (15.93%)
Mapped (bp)	94,159,422 (99.90%)
Properly mapped (bp)	92,800,646 (98.46%)
PE mapped (bp)	94,082,290 (99.82%)
SE mapped (bp)	154,264 (0.16%)
With mate mapped to a different chr	730,940 (0.78%)
With mate mapped to a different chr (mapQ ≥5)	659,900 (0.70%)
Initial bases on target (bp)	60,456,963
Initial bases on or near target (bp)	136,297,444
Total effective yield (Mb)	14,058.43
Effective yield on target (Mb)	9,638.81
Fraction of effective bases on target (%)	68.6%
Fraction of effective bases on or near target (%)	90.3%
Average sequencing depth on target	159.43
Bases covered on target (bp)	60,311,835
Coverage of target region (%)	99.8%
Fraction of target covered with at least 100 × (%)	63.6%
Fraction of target covered with at least 50 × (%)	85.2%
Fraction of target covered with at least 20 × (%)	95.6%
Fraction of target covered with at least 10 × (%)	98.2%
Fraction of target covered with at least 4 × (%)	99.3%
Total SNPs	127,172
Novel SNPs	791
Total InDels	14,713
Novel InDels	1,127
Gender	Male

### Prediction of the protein structure

**Figure 3:**
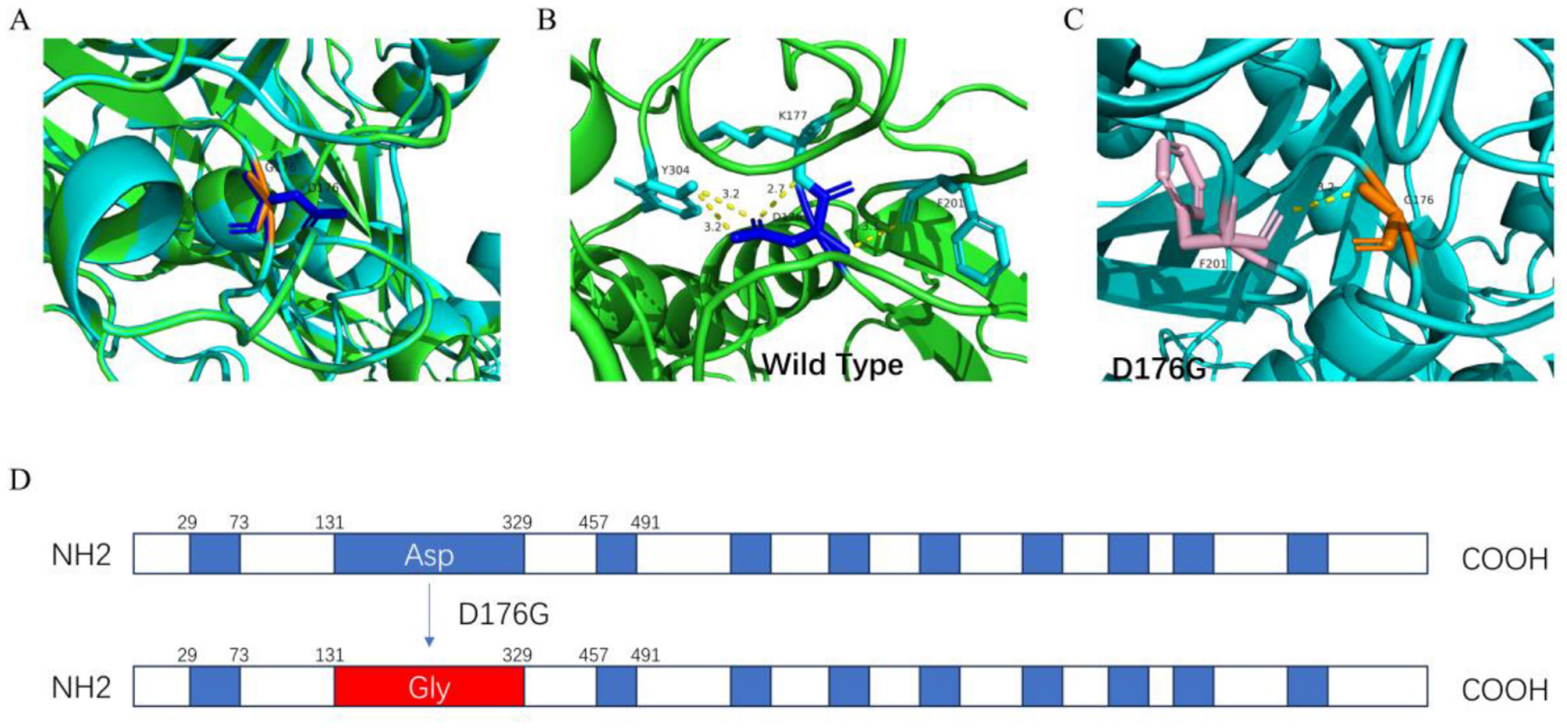
Three-dimensional (3D) rendering and structural analysis of both the wild-type and mutant (D176, G176) variants of the ITGB4 protein. **(A)** The 3D model of the ITGB4 protein, which was constructed and rendered using I-TASSER and PyMOL software. **(B)** The 3D structure of the ITGB4 in its wild-type configuration. **(C)** The 3D structure of the ITGB4 with the specified mutant genotype. **(D)** Schematic diagram of the mutation of aspartic acid to glycine at codon 176 in VWFA domain (131–329).

The predicted tertiary structures of both the wild-type and mutant ITGB4 proteins were obtained using the I-TASSER software, and their molecular structure was displayed using the PyMOL software A (Figure 3). The novel variant c.A527G in the *ITGB4* gene caused the 176th amino acid to change from aspartic acid to glycine and the polar amino acid to change to non-polar amino acid. This variant may locally affect the structure of the ITGB4 protein: wild-type D176 forms one hydrogen bond at a distance of 3.1 Å with F201, one hydrogen bond at a distance of 2.7 Å with K177, and two hydrogen bonds at a distance of 3.2 Å with Y304. However, the mutant G176 only forms one hydrogen bond with F201 at a distance of 3.2 Å. The reduction of hydrogen bonds between amino acids in the mutant protein and the transition of amino acids from polar to non-polar forms may affect the folding, stability, and solubility of proteins, as well as alter their interactions with other molecules, leading to a change in the protein structure and function and resulting in EB.

## Discussion

Here, we describe a novel variation at position D176G of the *ITGB4* gene in a family, characterized by a change in protein polarity and a decrease in hydrogen bonding. This was an autosomal dominant genetic variation caused by structural variations in the *ITGB4* gene, and Sanger's sequencing of genomic DNA confirmed the presence of this variation in the plaintiff and other affected family members.

Classical EB can be classified into four types: EBS, JEB, DEB, and KS (18). However, the variations that may exist in EB are complex, as variations in the same gene may be inherited in an autosomal dominant or recessive manner, and may lead to different clinical phenotypes. Thus, similar phenotypes in EBS and DEB may be dominant or recessive. For example, the mutated genes *KRT5* and *KRT14* in EBS and the mutated gene *COL7A1* in DEB can have dominant or recessive inheritance, whereas variations in JEB and KS are inherited in an autosomal recessive manner, e.g., the mutated genes *LAMB3, LAMC2, COL17A1, ITGA6, ITGB4*, and *ITGA3* in JEB (19, 20). A mutation in the *ITGB4* gene, which is responsible for producing the half-bridge integrin β4 protein, plays a crucial role in preserving the skin and epithelial integrity. This mutation is frequently associated with JEB with pyloric atresia (JEB-PA). JEB-PA is a rare, life-threatening, autosomal recessive disorder marked by the formation of blisters in the skin and mucous membranes. It is often associated with congenital defects in the gastrointestinal tract, which can result in significant mortality (21). In these cases, we noticed that the autosomal dominant genetic disease EB caused by heterozygous pathogenic variations in the *ITGB4* gene usually presented as mild symptoms. These symptoms include early onset nail malnutrition and possible mild skin blisters over time. In some cases, granulation tissue hyperplasia may also occur outside the dermis layer. In our research, we observed that patients clinically diagnosed with EB who had skin blisters, ulcers, and scabs carried the *ITGB4* variation, whereas those without clinical symptoms did not carry this variation. This indicates that the *ITGB4* gene variation in our family is also autosomal dominant inheritance, which is consistent with the report by Malovitski et al., Turcan et al. (14, 22). In the article published by scholar Turcan, they found a heterozygous missense variant c.G433T, p.D145Y in *ITGB4*, and their family patients presented with pachyonychia dystrophic nails and delayed mild limb blistering, in addition, some family members developed extracutaneous complications, such as external auditory canal. However, the patients in our study developed blisters on the surface of the skin, with the skin lesions being the shallowest and not involving the mucosa or extracutaneous complications. The variants p.D145Y and p.D176G are located in the VWFA domain of *ITGB4*, but their clinical symptoms differ greatly, and the specific mechanisms of these differences need further in-depth research.

Some *ITGB4* gene variations are located in the transmembrane domain. Owing to the composition of non-polar amino acids in the transmembrane domain, changes in the polarity of amino acid are highly likely to cause damage to the membrane anchoring function of integrin proteins. As a class of key transmembrane proteins, integrins are notably characterized by not only their ability to transmit signals across membranes but also their ability to regulate many complex functions in organisms such as cell adhesion, migration, and tissue repair (23, 24). We found a variation in the VWFA domain of *ITGB4*, which has attracted great interest from the scientific community since its discovery, owing to its various important cellular functions. These include basement membrane formation, cell migration, ligand binding, and signal transduction (25, 26). Puhm et al. (27) showed that the lack of VWFA domains has a significant impact on F15 biofilms. The LapA VWFA domain is described as necessary for hydrophilic surface binding reference (27). Our research first discovered a variation in the 176th position of the VWFA domain in *ITGB4*, which changes the amino acid from a polar amino acid to a non-polar amino acid. This may weaken its ability to form hydrogen bonds with other polar molecules, such as water molecules, and may affect protein solubility. Furthermore, we found that the hydrogen bonds between amino acids in the mutant protein changed from two to one, which may have locally affected the structure of the ITGB4 protein, affecting its folding, stability, and solubility, and altering its interactions with other molecules. This may have led to changes in protein function, such as possibly affecting the binding of laminin and E-cadherin, causing damage to the structure of the biofilm, disrupting the integrity of the skin and epithelium, and ultimately leading to the dissolution of the bullous epidermis. However, the potential molecular mechanism of the *ITGB4* c.A527G (D176G) mutation in JEB still remains unclear, and the relationship between genotype and phenotype needs further investigation (28).

Overall, this study outlines the autosomal dominant inheritance of JEB caused by mutations in the *ITGB4* gene, which is a novel missense variant in *ITGB4* A527G (D176G) and has been characterized by delayed limb blisters. It is necessary for future research to gain a deeper understanding of the genetic patterns and clinical manifestations of *ITGB4*-related JEB, and to reveal the molecular mechanisms through which *ITGB4* gene mutations lead to different clinical manifestations.

## Data Availability

The original contributions presented in the study are publicly available. This data can be found here: NCBI SRA, PRJNA1414680.

## References

[1] Zhou X Wang M Wang S Jiang X Li W. Identification of novel compound heterozygous ITGB4 mutations in a Chinese woman with junctional epidermolysis bullosa without pylori atresia but profound urinary symptoms: a case report and review of the literature. J Dermatol. (2021) 48:1780–5. doi: 10.1111/1346-8138.16104 34462954

[2] Masunaga T Niizeki H Yasuda F Yoshida K Amagai M Ishiko A. Splicing abnormality of integrin beta4 gene (ITGB4) due to nucleotide substitutions far from splice site underlies pyloric atresia-junctional epidermolysis bullosa syndrome. J Dermatol Sci. (2015) 78:61–6. doi: 10.1016/j.jdermsci.2015.01.016 25728941

[3] Yu Y Wang Z Mi Z Sun L Fu X Yu G . Epidermolysis bullosa in Chinese patients: genetic analysis and mutation landscape in 57 pedigrees and sporadic cases. Acta Derm Venereol. (2021) 101:adv00503. doi: 10.2340/00015555-3843 34046686 PMC9413781

[4] Luo C Yang L Huang Z Su Y Lu Y Yu D . Case report: a case of epidermolysis bullosa complicated with pyloric atresia and a literature review. Front Pediatr. (2023) 11:1098273. doi: 10.3389/fped.2023.1098273 37033187 PMC10076629

[5] Schumann H Kiritsi D Pigors M Hausser I Kohlhase J Peters J . Phenotypic spectrum of epidermolysis bullosa associated with alpha6beta4 integrin mutations. Br J Dermatol. (2013) 169:115–24. doi: 10.1111/bjd.12317 23496044

[6] Bergman R. Immunohistopathologic diagnosis of epidermolysis bullosa. Am J Dermatopathol. (1999) 21:185–92. doi: 10.1097/00000372-199904000-00015 10218683

[7] Fine JD Eady RA Bauer EA Bauer JW Bruckner-Tuderman L Heagerty A . The classification of inherited epidermolysis bullosa (EB): report of the third international consensus meeting on diagnosis and classification of EB. J Am Acad Dermatol. (2008) 58:931–50. doi: 10.1016/j.jaad.2008.02.004 18374450

[8] Hou P-C Wang H-T Abhee S Tu W-T McGrath JA Hsu C-K. Investigational treatments for epidermolysis bullosa. Am J Clin Dermatol. (2021) 22:801–17. doi: 10.1007/s40257-021-00626-3 34292508

[9] Has C Liu L Bolling MC Charlesworth AV El Hachem M Escámez MJ . Clinical practice guidelines for laboratory diagnosis of epidermolysis bullosa. Br J Dermatol. (2020) 182:574–92. doi: 10.1111/bjd.18128 31090061 PMC7064925

[10] Peters M Reber I Jagannathan V Raddatz B Wohlsein P Drogemuller C. DNA-based diagnosis of rare diseases in veterinary medicine: a 44 kb deletion of ITGB4 is associated with epidermolysis bullosa in Charolais cattle. BMC Vet Res. (2015) 11:48. doi: 10.1186/s12917-015-0366-0 25890340 PMC4351973

[11] Matyas M Miclea D Zaharie G. Case report: uncommon association of ITGB4 and KRT10 gene mutation in a case of epidermolysis bullosa with pyloric atresia and aplasia cutis congenita. Front Genet. (2021) 12:641977. doi: 10.3389/fgene.2021.641977 34306001 PMC8296908

[12] Chen F Wei R Deng D Zhang X Cao Y Pan C . Genotype and phenotype correlations in 441 patients with epidermolysis bullosa from China. J Eur Acad Dermatol Venereol. (2023) 37:411–9. doi: 10.1111/jdv.18692 36287101

[13] Dang N Klingberg S Rubin AI Edwards M Borelli S Relic J . Differential expression of pyloric atresia in junctional epidermolysis bullosa with ITGB4 mutations suggests that pyloric atresia is due to factors other than the mutations and not predictive of a poor outcome: three novel mutations and a review of the literature. Acta Derm Venereol. (2008) 88:438–48. doi: 10.2340/00015555-0484 18779879

[14] Malovitski K Meijers O Cohen-Barak E Bergman J Adir N Giladi M . Heterozygous variants in the integrin subunit beta 4 gene (ITGB4) cause autosomal dominant nail dystrophy. Br J Dermatol. (2022) 187:826–8. doi: 10.1111/bjd.21774 35822394

[15] Fu F Tao X Jiang Z Gao Z Zhao Y Li Y . Identification of germline mutations in East-Asian young never-smokers with lung adenocarcinoma by whole-exome sequencing. Phenomics. (2023) 3:182–9. doi: 10.1007/s43657-022-00062-1 37197646 PMC10110802

[16] Cheng WZ Wang WH Deng AP Dang X Liu C Wang XC . Identification of an LDLR variant in a Chinese familial hypercholesterolemia and its relation to ROS/NLRP3-Mediated pyroptosis in hepatic cells. J Geriatr Cardiol. (2023) 20:341–9. doi: 10.26599/1671-5411.2023.05.003 37397863 PMC10308174

[17] Dong XQ Qin PP Zhang D Zhang QY Qu Y Zhao L . A novel missense mutation in obscurin gene in a Chinese consanguineous family with left ventricular noncompaction. J Geriatr Cardiol. (2022) 19:531–8. doi: 10.11909/j.issn.1671-5411.2022.07.011 35975021 PMC9361159

[18] Laimer M Prodinger C Bauer JW. Hereditary epidermolysis bullosa. J Dtsch Dermatol Ges. (2015) 13:1125–33. doi: 10.1111/ddg.12774 26513070

[19] Has C Bauer JW Bodemer C Bolling MC Bruckner-Tuderman L Diem A . Consensus reclassification of inherited epidermolysis bullosa and other disorders with skin fragility. Br J Dermatol. (2020) 183:614–27. doi: 10.1111/bjd.18921 32017015

[20] Fine JD Bruckner-Tuderman L Eady RA Bauer EA Bauer JW Has C . Inherited epidermolysis bullosa: updated recommendations on diagnosis and classification. J Am Acad Dermatol. (2014) 70:1103–26. doi: 10.1016/j.jaad.2014.01.903 24690439

[21] Ko L Griggs CL Mylonas KS Masiakos PT Kroshinsky D. A nonlethal case of junctional epidermolysis bullosa and congenital pyloric atresia: compound heterozygosity in a patient with a novel integrin beta 4 gene mutation. J Pediatr. (2018) 193:261–4 e1. doi: 10.1016/j.jpeds.2017.09.023 29198538

[22] Turcan I Pasmooij AM van den Akker PC Lemmink H Halmos GB Sinke RJ . Heterozygosity for a novel missense mutation in the ITGB4 gene associated with autosomal dominant epidermolysis bullosa. JAMA Dermatol. (2016) 152:558–62. doi: 10.1001/jamadermatol.2015.5236 26817667

[23] Abe M Sawamura D Goto M Nakamura H Nagasaki A Nomura Y . ITGB4 missense mutation in a transmembrane domain causes non-lethal variant of junctional epidermolysis bullosa with pyloric atresia. J Dermatol Sci. (2007) 47:165–7. doi: 10.1016/j.jdermsci.2007.03.001 17512702

[24] Widhiati S Pradipta NK Purnomosari D Danarti R Anggraini A Palupi-Baroto R . Lethal Carmi syndrome (junctional epidermolysis bullosa-pyloric atresia) with a novel and recurrent compound integrin beta 4 heterozygous mutation: a case report. JAAD Case Rep. (2024) 50:75–8. doi: 10.1016/j.jdcr.2024.05.020 39070921 PMC11282936

[25] Tuckwell DS Humphries MJ. A structure prediction for the ligand-binding region of the integrin beta subunit: evidence for the presence of a von Willebrand factor A domain. FEBS Lett. (1997) 400:297–303. doi: 10.1016/S0014-5793(96)01368-3 9009218

[26] Colombatti A Bonaldo P. The superfamily of proteins with von Willebrand factor type A-like domains: one theme common to components of extracellular matrix, hemostasis, cellular adhesion, and defense mechanisms. Blood. (1991) 77:2305–15. doi: 10.1182/blood.V77.11.2305.bloodjournal77112305 2039815

[27] Puhm M Hendrikson J Kivisaar M Teras R. Pseudomonas putida biofilm depends on the vWFa-domain of LapA in peptides-containing growth medium. Int J Mol Sci. (2022) 24:23. doi: 10.3390/ijms23115898 35682576 PMC9180339

[28] Ying W. Phenomic studies on diseases: potential and challenges. Phenomics. (2023) 3:285–99. doi: 10.1007/s43657-022-00089-4 36714223 PMC9867904

